# Case Report: Acute hydrotherapy with super-saturated hydrogen-rich water for ankle sprain in a professional athlete

**DOI:** 10.12688/f1000research.22850.1

**Published:** 2020-04-08

**Authors:** Dejan Javorac, Valdemar Stajer, Sergej Ostojic

**Affiliations:** 1 FSPE Applied Bioenergetics Lab, University of Novi Sad, Novi Sad, 21000, Serbia; 2 Faculty of Health Sciences, University of Pecs, Pecs, H-7621, Hungary

**Keywords:** molecular hydrogen, ankle sprain, hydrotherapy

## Abstract

**Background:** The traditional treatment of soft tissue injuries consists of the RICE protocol – rest, ice, compression, and elevation, followed for up to 72 hours after a trauma. Although designed as an immediate therapy to reduce inflammation that occurs after an acute injury, the RICE protcol might not be the best way to promote healing due to limiting blood flow. Molecular hydrogen (H _2_) has recently been put forward as a possible adjuvant treatment in musculoskeletal medicine, yet limited data are available concerning its effectiveness as a first-aid intervention.

**Case report:** We report here a case of an elite professional athlete who suffered a grade II ankle sprain, and who subsequently received six sessions of ankle and foot hydrotherapy (e.g. 30-min at every four hours) with super-saturated hydrogen-rich water during the first 24 hours post-injury. The pain VAS self‐completed by the patient dropped from 50 points (moderate pain) at baseline (immediately after injury) to 20 points (mild pain) at 24-h follow-up. Ankle swelling dropped by 2.8% and dorsiflexion range of movement improved by 27.9% from baseline to follow-up, respectively.

**Conclusions: **Our case has indicated that an acute multi-session hydrotherapy with hydrogen-rich water might be a helpful treatment in terms of pain, swelling reduction and regaining range of motion after an ankle sprain.

## Introduction

Soft tissue injuries (STIs) remain among the most prevalent traumata in musculoskeletal medicine ^1^. Sprains, strains and contusions are common STIs that often occur during sport and exercise activities ^2^. STIs usually require immediate treatment to reduce inflammation, bleeding and damage within the injured tissue (e.g. muscle, tendon, ligaments, joint), with management options typically comprise a break from the activity that caused an injury, and different physical therapy procedures. Applying rest, ice, compression, and elevation (RICE) therapy is referred as a long standard first-aid treatment protocol for STIs ^3^. However, insufficient evidence appears to be available to determine the relative effectiveness of RICE therapy for specific STIs ^4^. In particular, RICE therapy might not be the best way to promote tissue healing due to limiting blood flow, and alternative methods and techniques are advocated to help manage STIs ^5^. Among others, molecular hydrogen (H _2_) has recently been put forward as a possible adjuvant treatment in musculoskeletal medicine ^6^. Addition of oral and topical H _2_ intervention to RICE protocol was effective to reduce inflammation and augment range-of-motion recovery in athletes who suffered a soft tissue injury ^7^. However, no data are available concerning the effectiveness of hydrogen when applied as an individual first-aid intervention. This case report illustrates the efficacy and safety of acute multi-session hydrotherapy with super-saturated hydrogen-rich water for ankle sprain in a professional male athlete.

## Case report

### Patient information

We are reporting here the case of a 29 year old male Caucasian professional football athlete who suffered a sport-related ankle sprain in April 2019. The injury occurred during a regular exercise session on artificial turf as an inversion sprain accompanied by plantar flexion. The patient was immediately evaluated by a sports medicine specialist who confirmed the category and the degree of injury (grade II ankle sprain) by physical examination. The patient was an apparently healthy young men (age 29 years, weight 77.0 kg, height 184.0 cm, professional experience 11 years), with no history of ankle sprain (or other lower extremity injuries) in the past 6 months, and no cardiometabolic or other musculoskeletal disorders. Written informed consent was obtained from the patient in accordance with the Declaration of Helsinki, and study protocol approved by the local IRB at the FSPE Applied Bioenergetics Lab at the University of Novi Sad (A14-2019).

### Clinical findings

**Figure 1.**
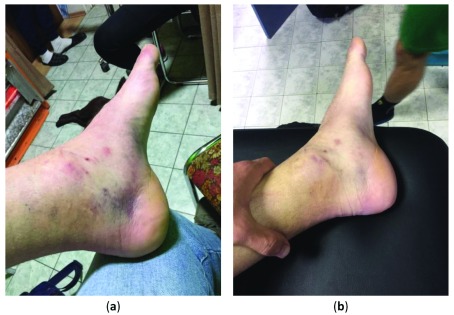
An image of sprained ankle: ( **a**) Immediately after the injury; ( **b**) At 24-h follow-up after six 30-min sessions of hydrotherapy with super-saturated hydrogen-rich water.

At the initial examination immediately after the injury, right ankle was painful, swollen and warm, and had increased laxity on testing ( Figure 1, Panel a). Self-completed visual analog scale (VAS) score for pain was 50 points (moderate pain). Figure-of-eight method of measuring ankle joint swelling at the injured ankle revealed 56.5 cm, with weight-bearing lunge test (WBLT) showing 43.1 mm. The patient demonstrated a diminished ability to bear weight.

### Hydrogen therapy

Hydrotherapy with super-saturated hydrogen-rich water was used as an exclusive treatment (besides rest) with the main aim to reduce symptoms and signs of acute ankle sprain, and it was hoped to speed up the acute recovery. Super-saturated hydrogen-rich water was produced by putting a magnesium-producing formulation (10 g) into a 3-L stationary whirlpool with tap water of neutral temperature (20°C). Hydrogen was produced by a following reaction: Mg + H _2_O → H _2_ + Mg(OH) _2_, with concentration of hydrogen in a whirlpool ~ 8 ppm. The intervention was provided by HRW Natural Health Products Inc. (catalogue number 6-27843; New Westminster, BC, Canada). Throughout the 24 hours after the ankle sprain, the participant received six 30-min ankle baths (e.g. one hydrotherapy every 4 hours), with the first session given immediately after an initial examination (~ 60 min after the injury). During each session, the foot and ankle of injured leg were immersed in a stationary whirlpool. All hydrotherapies were formulated and supervised by a health care professional.

### Follow-up and outcomes

At the 24-h follow-up examination ( Figure 1, Panel b), VAS score for pain dropped to 20 points (mild pain), with ankle circumference decreased to 54.9 cm; WBLT improved to 55.0 mm. The patient reported no side effects of hydrogen intervention (e.g. pain, cramps, tingling, discoloration of skin, burning, itching, rash), as evaluated with open-ended questionnaire administered at the end of each session of hydrotherapy, and at 24-h follow-up.

## Discussion

This case report suggests the beneficial effects of hydrotherapy with hydrogen-rich water as a possible treatment to decrease pain and swelling in a professional athlete with moderate-degree ankle sprain. A number of recent animal and human studies evaluated the efficacy of H _2_ in musculoskeletal medicine. H _2_ appears to be effective in tackling disuse muscle atrophy in rats ^8^, Duchenne muscular dystrophy in mice ^9^, bone loss induced by modeled microgravity ^10^ or ovariectomy-induced osteoporosis in rats ^11^, and mitigate disease activity in patients with rheumatoid arthritis ^12^. For acute injuries, hydrogen-rich saline (1 ml/100 g) seems to be beneficial in attenuating muscle damage in a rat model of skeletal muscle injury induced by 3-h tourniquet occlusion and 4-h reperfusion ^13^. Our group reported advantageous effects of 2-week administration of topical and oral H _2_ (added to the RICE therapy) on plasma viscosity and functional recovery in a randomized controlled trial with professional athletes who suffered STIs ^7^. In this case report, we found that acute hydrotherapy with super-saturated hydrogen-rich water might be beneficial when used as the exclussive first-aid treatment in specific STIs, such as ankle sprain. H _2_ positively affected STI-induced signs and symptoms at 24-h follow up perhaps due to its antioxidant, anti-inflammatory and anti-apoptotic effects ^14^. Due to its small size and higher-grade diffusibility ^6^, topical hydrogen could be easily transported to hard-to-reach tissues, including areas of injury that are often characterized by low drug penetrability ^15^. Specifically, H _2_ might prevent (or offset) the generation of toxic compounds that occur after initial injury due to subsequent cell damage and tissue hypoxia, often referred as a secondary injury ^16^.

This professional athlete received a rather intensive treatment, with H _2_ hydrotherapy applied in several recurrent episodes throughout the first 24 hours post-injury, a pattern that might be highly applicable to an athlete looking for an accelerated and efficacious strategy in STIs management and recovery ^3^. This treatment dynamics appear to be comparable to the traditional acute RICE protocol for STIs medical care in terms of frequency (e.g. number of sessions per day) and duration of intervention (e.g. length of individual session) ^4^. On the other hand, acute H _2_ hydrotherapy may be superior to RICE protocol due to improved blood flow (as implied here by a decreased swelling at follow-up), while the ice component of RICE reduces blood flow to the injured area and delays healing ^5^. To confirm this hypothesis, future case series should compare two interventions in a double-blind, parallel-group randomized controlled design, by evaluating peripheral circulation at the site of injury.

Due to the fact that hydrogen in water tends to evaporate over time ^17^, hydrogen-rich water for every session was prepared fresh and administered for 30 min. A report have shown that gaseous hydrogen remains detained in water exposed to air for up to 2 hours before its concentration drops below a therapeutical level (e.g. < 1 ppm) ^18^. The super-saturated hydrogen-rich water used here (8 ppm) perhaps provides an advantage of supraphysiological dosages of H _2_ administered in a time-optimized manner. However, how H _2_ concentration changes during the session of hydrotherapy remains unknown. Despite that, topical H _2_ hydrotherapy yielded no side effects in our patient, confirming an affirmative safety record for H _2_ reported by others [for review see Ref. 16].

Several limitations must be considered when the study findings are interpreted. First, we report a relatively short period of the intervention and post-injury assessment (e.g. 24 hours) while no medium- and long-term efficacy and safety of topical hydrogen were evaluated. Second, only limited compendium of clinician- and patient-reported outcomes were analyzed, while no biomarkers of tissue injury or inflammation were employed, and a possible mechanism of hydrogen action remains unknown. Finally, it remains open to question how topical H _2_ positively affects the acute recovery of other STIs with different location, etiology and severity.

## Conclusions

Even though our results are based on a single-patient report, our case has indicated that an acute multi-session hydrotherapy with hydrogen-rich water might be a safe and helpful treatment in terms of pain, swelling reduction, and regaining the range of motion after an ankle sprain. We suggest a closer monitoring of the efficacy and safety of topical H _2_ therapy use in different musculoskeletal injuries on a larger similar case series.

## Consent

Written informed consent was obtained from the patient for the publication of this case report, including any associated images.

## Data availability

### Underlying data

All data underlying the results are available as part of the article and no additional source data are required.
