# Supplementary material for: Case Report: Acute hydrotherapy with super-saturated hydrogen-rich water for ankle sprain in a professional athlete
Source: F1000Res. 2020 Apr 8;9:245. [Version 1] doi: 10.12688/f1000research.22850.1 (PMC7194471; doi:10.12688/f1000research.22850.1)
Supplement: Peer review history

# Peer review: Reviewer response for version 1

**DOI:** 10.5256/f1000research.25228.r62120

**Published:** 2020-04-30

**Authors:** Cheong Hwa Ooi

**Affiliations:** 1 Lifestyle Science Cluster, Advanced Medical and Dental Institute, Universiti Sains Malaysia, Kepala Batas, Malaysia

The authors of this case report argue that there is insufficient evidence on the effectiveness of RICE therapy for specific soft tissue injuries. Hence, further development of the intervention for soft tissue injuries is warranted. With that, they presented a case of a professional footballer who suffered a grade II ankle sprain and subsequently treated with six 30-minute foot immersion in hydrogen-rich water (3 L, ~8 ppm). The author concluded that the treatment might be safe and helpful in reducing pain and swelling, and regaining the range of motion after an ankle sprain. 

 It seems that it is the left ankle, as shown in Figure 1, but the authors reported it as right ankle sprain in the text. Further clarification is needed. The treatment was given every 4 hours throughout the 24 hours after injury, and it is reasonable to assume that the nighttime sleep would have been disrupted. Does it represent a feasible and ideal therapy for the athlete? The water temperature for the foot/ankle bath was 20°C, how does adding hydrogen-producing magnesium tablets into it help the blood flow? Further explanation of the mechanism for improved blood flow after the treatment is relevant to the understanding of hydrogen medicine in soft tissue injury.

Are enough details provided of any physical examination and diagnostic tests, treatment given and outcomes?

Yes

Is the case presented with sufficient detail to be useful for other practitioners?

Yes

Is sufficient discussion included of the importance of the findings and their relevance to future understanding of disease processes, diagnosis or treatment?

Yes

Is the background of the case’s history and progression described in sufficient detail?

Yes

Reviewer Expertise:

Exercise Physiology

I confirm that I have read this submission and believe that I have an appropriate level of expertise to confirm that it is of an acceptable scientific standard.

# Response

**Published:** 2020-04-30

**Authors:** Sergej Ostojic

**Affiliations:** University of Novi Sad, Serbia

Thank you for your valuable comments and notes.

 We confirmed that the right ankle was injured yet we depicted here a mirror image.

 Also, the intensive hydrogen treatment might disrupt sleeping time but that's the case with all intensive measures for acute ankle injuries since the treatment requires immediate and intensive care during the firts 24 to 48 h do to specific pathophysiology.

 Further studies are highly warranted to evaluate the possible effects of hydrogen on blood flow, a factor that might contribute to its mechanism(s) of action.

# Peer review: Reviewer response for version 1

**DOI:** 10.5256/f1000research.25228.r62119

**Published:** 2020-04-21

**Authors:** Steven R. Brenner

**Affiliations:** 1 Department of Neurology and Psychiatry, Saint Louis University School of Medicine, St. Louis, MO, USA

I thought there was good documentation of the injury including physical examination, photographs and pain scales.

 The application of hydrogen water as treatment with reference to schedule and treatment application was well documented and should be repeatable.

 The ankle injury was to a professional athlete. Was the athlete able to return to athletic competition as soon utilizing the hydrogen water method as would be possible using the more traditional “RICE”, approach to treatment of ankle injuries, primarily sprained ankle injury?

Are enough details provided of any physical examination and diagnostic tests, treatment given and outcomes?

Yes

Is the case presented with sufficient detail to be useful for other practitioners?

Yes

Is sufficient discussion included of the importance of the findings and their relevance to future understanding of disease processes, diagnosis or treatment?

Yes

Is the background of the case’s history and progression described in sufficient detail?

Yes

Reviewer Expertise:

Neurology

I confirm that I have read this submission and believe that I have an appropriate level of expertise to confirm that it is of an acceptable scientific standard.

# Response

**Published:** 2020-04-30

**Authors:** Sergej Ostojic

**Affiliations:** University of Novi Sad, Serbia

Thank you for your comment.

 We haven't been able to evaluate post-injury recovery in terms of return to athletic competition due to the nature of this study, However, this issue requires an additional trial since this aspect might be of great importance for professional athletes who usually strive for re-getting to competition as soon as possible.
